# Comparison of Three Different Surgical Fixation Techniques in Pediatric Forearm Double Fractures

**DOI:** 10.7759/cureus.16931

**Published:** 2021-08-06

**Authors:** Hakan Zeybek, Sefa Akti

**Affiliations:** 1 Department of Orthopaedics and Traumatology, Izmir Katip Çelebi University, İzmir Atatürk Training and Research Hospital, İzmir, TUR; 2 Department of Orthopaedic and Traumatology, Aksaray University Education and Research Hospital, Aksaray, TUR

**Keywords:** forearm double fracture, pediatric fractures, elastic stable intramedullary nailing, open reduction internal fixation, hybrid fixation

## Abstract

Introduction

Although forearm fractures are one of the most common fractures in children, controversy remains about the optimal fixation technique in the surgical treatment of these fractures. The aim of this study was to compare the results of pediatric patients with a forearm double fracture who underwent open reduction and internal fixation (plate-screw group), elastic intramedullary nailing to both bones (ESIN), and hybrid fixation (the radius or ulna is fixed with the help of ESIN and the other forearm bone is fixed using plate-screw).

Methods

A retrospective scan was made of the records of 51 patients with forearm double diaphyseal fractures (19 plate-screw, 18 ESIN, and 14 hybrid fixation). Comparisons were made in respect of the duration of surgery, length of the incision, duration of follow-up, time to union, functional results, and complication rates.

Results

The study results showed that the surgical incision length and operating time in the hybrid group were statistically significantly shorter than in the plate-screw group (p<0.05). No statistically significant difference was determined between the three groups in respect of the functional results (p>0.05).

Conclusion

When the hybrid fixation technique was compared with other methods, the results were equal or better in terms of surgery time, incision length, time to union, and complications. Therefore, this technique can be used as an effective and reliable method in appropriate pediatric forearm diaphyseal double fractures.

## Introduction

Forearm fractures are among the most common fractures in children, and there has been observed to have been increasing incidence in the last 10 years [1]. Forearm double diaphyseal fractures constitute 5.4% of all pediatric fractures under the age of 16 years, with boys are affected almost twice as often as girls [2]. In the treatment of forearm double diaphyseal fractures in children, inadequate fracture reduction, instability of the fracture, polytrauma, open fracture, loss of fracture reduction, and compartment syndrome constitute surgical indications [3].

Forearm double fractures are considered intra-articular fractures because they impair the rotational function of the wrist and elbow joints, and the aim of surgical treatment is to restore axial and rotational stability and to provide a functional range of motion [4, 5]. The most common surgical methods for forearm double diaphyseal fractures are intramedullary elastic nail application (ESIN) and open reduction plate-screw fixation, which are considered the best options [6, 7]. The ESIN method has advantages such as better cosmetic appearance, less stripping of the bone periosteum, and small surgical incisions [8]. However, complications such as irritation of the skin due to the implant, migration of the implant, compartment syndrome, and a relatively high rate of failed fixation and pseudoarthrosis compared to plate screw fixation, and delayed union have been reported [8, 9]. Plate-screw fixation provides anatomic reduction and more stable fixation, with almost complete restoration of forearm rotational movements. However, this approach has disadvantages such as requiring extensive soft tissue dissection, periosteal damage during plate application, and an increased risk of re-fracture after the plate removal. [7, 10]. 

In the hybrid fixation method, in which the radius or ulna is fixed with the help of ESIN and the other forearm bone is fixed using plate-screw, the anatomic reduction and relatively strong fixation of the bone that is applied with plate-screw fixation is achieved, while the ESIN fixation of the other bone is also provided. The hybrid fixation method reduces the amount of soft tissue dissection required during surgery and decreases the potential risk of re-fracture developing after implant removal [11].

There are few studies in the literature comparing these three methods in pediatric forearm double diaphyseal fractures [11]. Therefore, the aim of this study was to compare the radiological and functional results of pediatric patients with a forearm double fracture who underwent plate-screw, ESIN, and hybrid fixation. It was hypothesized that in the hybrid fixation method, better radiological, clinical, and functional results would be obtained with lower complication rates compared to other surgical methods.

## Materials and methods

Approval for the study was granted by the Local Ethics Committee and informed consent was obtained from the parents of all the patients. Following a retrospective scan of the digital and printed medical records in our hospital archives, 60 patients between the ages of six and 16 years who were operated on for forearm double diaphyseal fracture between 2015 and 2020 were included in the study. All surgery was operated by two senior surgeons of orthopedics.

The study inclusion criteria were: a) mid-third diaphyseal fracture, b) >10° of angulation or >30° of malrotation after closed reduction, c) six to 16 years of age, d) no pre-operative neurovascular injury, and e) a follow-up period of >15 months. The study exclusion criteria were defined as 1) ipsilateral or contralateral other upper extremity fracture/dislocation, 2) pathological fractures, 3) open fractures, 4) fractures with neurovascular injury 5) concomitant vital organ damage, and 6) complex forearm fractures (Monteggia fracture, Galeazzi fracture, intra-articular elbow or wrist fracture). A total of nine patients were excluded from the evaluations; postoperative radiographs were not available in four cases, accompanying humerus fracture in one, bilateral upper extremity forearm fractures in two, preoperative neurovascular injury in one, and one patient had multiple fractures and was followed up in the intensive care unit. Thus, the study was conducted on 51 patients.

**Figure 1:**
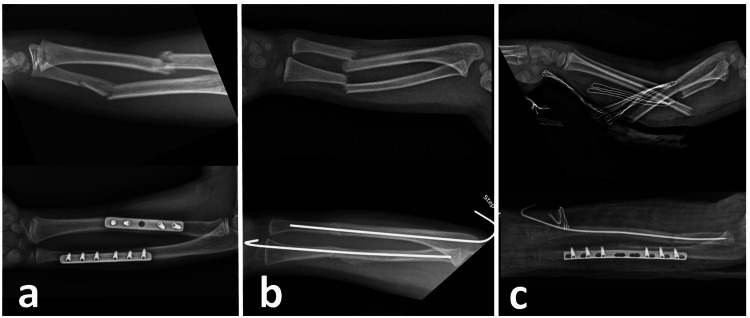
Pre-operative and post-operative follow-up radiographs of three types of fixation techniques. a) A-11-year-old boy with forearm double fractures treated with plate-screw fixation b) A-8-year-old girl with forearm double fractures underwent ESIN fixation. c) A-10-year-old boy with forearm double fractures operated with hybrid fixation.

The written and digital records of all the patients were reviewed. A record was made for each patient of age, gender, side of the fractured extremity, type of fracture, mechanism of injury, and time from trauma to surgery. Perioperatively, the duration of surgery, the length of the incision made, the time to union of each bone, the follow-up period, complications, and the functional results during follow-up were determined. Representative cases from three groups are shown in Figure 1. The consent had been obtained from the parents for possible publication of figures.

Longarm splint fixation was applied to all patients for three weeks after surgical treatment. Dressings were checked every other day, and prophylactic antibiotic therapy and pain control was administered when necessary. Physiotherapy was started as early as possible. The radiological and functional results of the patients were followed up at regular post-operative intervals.

The functional results at the final follow-up examination were evaluated according to the criteria of Price et al. as excellent (no complaints with compulsive physical activity and/or loss of pro-supination <10°), good (mild complaints and/or loss of forearm rotation 11-30° with challenging physical activity), fair (subjective complaints occurring in daily activities and/or loss of 30-90° forearm rotation) and poor (other results) [12]. Loss of rotation in the operated forearm was determined by comparing it with rotation in the intact contralateral forearm.

Pre-operative radiographs of the fractures were classified according to the Orthopedic Trauma Association (OTA) forearm diaphyseal fracture classification system [13]. Fracture union was accepted as the presence of callus in at least three cortices observed on the anteroposterior and lateral radiographs. As defined by Schmittenbecher et al., no union after the post-operative third month was considered “delayed union”, and no union after six months was considered “pseudoarthrosis” [14].

Data obtained in the study were analyzed statistically using IBM Corp. Released 2011. IBM SPSS Statistics for Windows, Version 20.0. Armonk, NY: IBM Corp. Categorical data were expressed as frequency and percentage, and continuous data as mean and standard deviation values. The conformity of the data to normal distribution was evaluated with the Shapiro Wilk test and scatter plots. The Chi-square test was used to compare categorical data. In the comparisons of continuous data between groups, ANOVA or the Kruskal Wallis test was applied. A value of p<0.05 was considered statistically significant.

## Results

The distribution of age, gender, fracture classification, affected side, and mechanism of injury according to the type of surgery performed is summarized in Table 1. There was no statistically significant difference between the groups in respect of gender, injury site, fracture type, injury mechanism, and time to surgery (p>0.05).

**Table 1:** Comparison of the techniques in respect of some preoperative diagnosis characteristics ^a^:Kruskal Wallis Test, ^b^:chi-square test, *: p<0.05

Characteristics	Plate-Screw Fixation	ESIN Fixation	Hybrid Fixation	p
Number of patients	19	18	14	
Age at surgery (years)	11.00±2.26	10.11±2.37	8.57±2.24	
Gender (n,%)				
Female	11 (57.9)	10 (55.6)	9 (64.3)	0.879^b^
Male	8 (42.1)	8 (44.4)	5 (35.7)	
Side of injury (n,%)				
Left	11 (57.9)	5 (27.8)	5 (35.7)	0.157^b^
Right	8 (42.1)	13 (72.2)	9 (64.3)	
AO classification (n,%)				
22-A	11 (57.9)	8 (44.4)	7 (50.0)	0.878^b^
22-B	6 (31.6)	6 (33.3)	5 (35.7)	
22-C	2 (10.5)	4 (22.2)	2 (14.3)	
Injury mechanism (n,%)				
Fall accident	17 (89.5)	15 (83.3)	11 (78.6)	0.879^b^
Traffic accident	2 (10.5)	3 (16.7)	2 (14.3)	
Other causes	0 (0.0)	0 (0.0)	1 (7.1)	
Injury to surgery time (days)	1.16±0.60	1.39±0.50	1.64±0.93	0.133^a^

Standard surgical procedures were followed for the surgical methods applied to the patients (4, 6). In the hybrid treatment group, a plate screw was applied to the displaced bone, while ESIN was applied to the non-displaced bone. Plate-screw was applied to the radius in 4 patients, and ESIN to the ulna, and ESIN was applied to the radius in 4 patients, and plate-screw to the ulna. When all three techniques were compared, a significant difference was observed in terms of the mean surgery time. The mean operative time was found to be significantly longer in the patients who underwent plate-screw fixation (65.95±6.73 minutes) compared to ESIN (50.28±4.82 minutes) and hybrid (51.36±4.75 minutes) fixation (p<0.05). There was no statistically significant difference between the ESIN fixation group and the hybrid fixation group (p>0.05). There was a statistically significant difference in incision length between the three techniques. The mean incision length in patients who underwent plate-screw (13.84±2.57 cm) fixation was significantly longer than that of patients who underwent ESIN (3.22±0.88 cm) and hybrid fixation (8.79±2.64 cm) (p<0.05). The mean incision length of the hybrid fixation technique was also significantly longer than in the ESIN fixation (p<0.05).

The mean follow-up period was 22.95±2.76 weeks in the plate-screw group, 22.11±3.25 weeks in the ESIN group, and 23.79±2.04 weeks in the hybrid fixation group, with no statistically significant difference observed between the groups (p>0.05). Time to union of the ulna was 8.58±2.59 weeks in the plate-screw group, 10.41±2.35 weeks in the ESIN group, and 9.79±1.48 weeks in the hybrid fixation group. For the radius, the time to union was 9.53±2.48 weeks in the plate-screw group, 11.18±2.46 weeks in the ESIN group, and 9.79±1.48 weeks in the hybrid fixation group. When the three techniques were compared in terms of the union times of the radius and ulna, no statistically significant difference was found (p>0.05)

The minimum follow-up period of all patients included in the study was 15 months. According to the criteria defined by Price et al., there was no statistically significant difference between the 3 techniques in the results obtained at the final follow-up examination (p>0.05) [12]. Although the proportion of patients with excellent and good results in the hybrid group (78.6%) was slightly less than in the other two groups (83.3% and 83.3%), the difference was not statistically significant (p>0.05) (Table 2).

**Table 2:** Comparison of the techniques in respect of operative characteristics ^a^:Kruskal Wallis test, ^b^:ANOVA, ^c^:Chi-square test,  ^d^:Mann Whitney U with Bonferroni correction, *: p<0.05

	Plate-Screw Fixation^1^ n=19	ESIN Fixation^2^ n=18	Hybrid Fixation^3^ n=14	p	post-hoc^d^
Duration of surgery (min)	65.95±6.73	50.28±4.82	51.36±4.75	<0.001^a.*^	1>2-3
Length of incision (cm)	13.84±2.57	3.22±0.88	8.79±2.64	<0.001^a.*^	1>3>2
Follow-up period (weeks)	22.95±2.76	22.11±3.25	23.79±2.04	0.248^b^	-
Union time (weeks)					
Radius	8.58±2.59	10.41±2.35	9.79±1.48	0.054^b^	-
Ulna	9.53±2.48	11.18±2.46	10.21±2.01	0.120^b^	-
Functional outcome, (n,%)					
Excellent	11 (57.9)	10 (55.6)	7 (50.0)	0.960^c^	-
Good	5 (26.3)	5 (27.8)	4 (28.6)		
Fair	3 (15.8)	2 (11.1)	2 (14.3)		
Poor	0 (0.0)	1 (5.6)	1 (7.1)		
Rate of excellent and good (%)	15 (83.3)	15 (83.3)	11 (78.6)	0.925^c^	

Complications were observed at a rate of 26.3% in the plate-screw group, 33.3% in the ESIN group, and 26.6% in hybrid fixation patients, with no statistically significant difference between the groups (p>0.05). Superficial infection developed in 3 (15.8%) patients, radial nerve damage in 1 (5.3%), and refracture in 1 (5.3%) of the patients applied with plate-screw fixation. Superficial infections were treated with antibiotics and daily dressings. One patient who developed radial nerve palsy was followed up and the neuropraxia resolved spontaneously after 3 months. In 1 patient, a fracture occurred in the 3rd week after implant removal due to a fall on the arm, and this was treated surgically.

In the ESIN group, superficial infection developed in 3 patients (16.7%), soft tissue irritation in 2 patients (11.1%), and pseudoarthrosis in 1 patient (5.6%). Superficial infections were treated with antibiotics and daily dressings. In cases with soft tissue irritation, the problem was caused by the ESIN implant protruding from the entry points to the skin, and the complaints of the patients disappeared with the removal of the implants after the union. Delayed-union that developed in 1 patient was due to insufficient fixation. This 14-year-old male patient was followed up, and the union was observed in the postoperative 19th week and no further intervention was required. Superficial infection developed in 3 patients (21.4%) and soft tissue irritation in 1 patient (7.1%) in the hybrid fixation group. Superficial infections were treated with antibiotics and daily dressings. In cases with soft tissue irritation, the complaints disappeared with the removal of the ESIN implant (Table 3).

**Table 3:** Comparison of the techniques in respect of postoperative complications ^c^: Chi-square test

	Plate-Screw Fixation^1^ n=19	ESIN Fixation^2^ n=18	Hybrid Fixation^3^ n=14	p^c^
Superficial infection (n,%)	3 (15.8)	3 (16.7)	3 (21.4)	^-^
Radial nerve palsy (n,%)	1 (5.3)	0 (0.0)	0 (0.0)	^-^
Soft tissue irritation (n,%)	0 (0.0)	2 (11.1)	1 (7.1)	^-^
Refracture (n,%)	1 (5.3)	0 (0.0)	0 (0.0)	-
Delayed-union (n,%)	0 (0.0)	1 (5.6)	0 (0.0)	^-^
Total (n,%)	5 (26.3)	6 (33.3)	4 (28.6)	0.893

## Discussion

Forearm double fractures are among the most common injuries among children and adolescents [15]. While most pediatric forearm double fractures can be successfully treated with closed reduction and casting, closed reduction may not be sufficient in some cases and surgical reduction and fixation may be required [6, 16]. Despite the trend among surgeons for a less aggressive approach to pediatric fractures, an increasing number of surgeons prefer surgical treatment for pediatric forearm diaphyseal fractures [3]. However, the optimal surgical fixation method to be preferred in these patients remains a matter of controversy. Until recently, ESIN fixation was widely used in pediatric double forearm fractures. The advantages of ESIN fixation are the use of small incisions, shorter surgical time, and minimal dissection at the fracture line [17]. Despite this, high complication rates related to the ESIN fixation technique have been reported in recent studies. These can be listed as delayed union, pseudoarthrosis of the ulna, infection, skin irritation from the implant, migration or failure of the implant, tendon injury, and compartment syndrome [18, 19]. It has been shown that delayed union of the ulna and pseudoarthrosis of the ulna are more common than previously thought in cases of pediatric forearm fractures with ESIN fixation [14, 20, 21]. Ogonda et al. reported that in patients who underwent anterograde ESIN fixation to the ulna, the frequency of delayed ulnar union or pseudoarthrosis was high, as the fracture line was distracted, whereas patients who underwent retrograde ESIN fixation to the radius developed union problems less frequently compared to the ulna due to compression of the fracture line [20]. Total complication rates have been reported to be between 16.4% and 50.0% in pediatric patients applied with ESIN for forearm diaphyseal fractures [18, 22]. In the current study, this rate was found to be 33.3%.

In the open reduction and internal fixation technique, after two separate incisions and revealing the fractures in both bones, adequate reduction, and appropriate implant placement are essential. This method provides a potential opportunity to obtain good functional results by reconstructing the radial bow, providing axial and rotational control of reduction, and regaining the range of motion of the forearm [6, 23]. One of the disadvantages of plate-screw fixation is that non-union may result from extensive soft tissue dissection and stripping of the bone periosteum to provide adequate exposure [10]. While complications of plate-screw application have been reported at rates of 28% and 33% in pediatric forearm diaphyseal fractures, this rate in the current study was 26.3% in patients who underwent plate-screw fixation [22, 24].

Hybrid fixation was applied in order to minimize some disadvantages of ESIN fixation and to provide some advantages of plate screw fixation. In this method, ESIN fixation was applied to the first bone to be reduced in forearm double diaphyseal fractures in children, while the other was applied with plate-screw fixation. Hybrid fixation has advantages over both ESIN fixation and plate screw fixation. These include making a large incision in only one bone, less soft tissue dissection, the placement of stress-shielding implants in only one bone, and less use of potentially irritating implants. Zheng et al. reported a shorter incision and surgery time in patients who underwent hybrid fixation compared to plate-screw, and fluoroscopy and post-operative immobilization time were found to be shorter compared to the ESIN group [11]. Zhu et al. found that the surgical time was shorter in the patients who had hybrid fixation compared to patients who were applied plate-screw, and a longer fluoroscopy time was reported [25]. In the current study, the surgical incision length and surgical time were determined to be significantly shorter in the hybrid group than in the plate-screw group, and these findings were consistent with the literature. Therefore, hybrid fixation can be recommended as a successful surgical treatment option in pediatric forearm diaphyseal fractures. Feng et al. determined that patients who underwent ESIN fixation had significantly delayed union of the ulna compared to the group that underwent hybrid fixation [26]. When Zheng et al. evaluated the rate of union of the ulna at the 3rd month, it was found to be less in the ESIN fixation group than in the hybrid and plate-screw groups [11]. In the current study, although the mean time to union of the radius and ulna (10.41±2.35 weeks and 11.18±2.46 weeks, respectively) was observed to be slower in the ESIN group, the difference between the groups was not statistically significant.

Functional results reported in the literature have shown no statistically significant difference between hybrid groups and ESIN or plate-screw applied groups. [11, 25, 26, 27]. In the current study, no significant difference was determined between the groups in respect of functional outcomes, consistent with the above-mentioned studies (p>0.05). Similarly, no statistically significant difference was determined between the three fixation groups in respect of total complication rates (p>0.05). When all these findings are evaluated together, the hybrid fixation technique applied in pediatric forearm double fractures can be seen as a safe and acceptable surgical method.

This study had some limitations, primarily the restrospective design and the low number of cases, which weaken the statistical analysis power of the study. Attempts to determine standardized assessments of bone union in a retrospective study are very limited. Stronger clinical data can be obtained with future larger scale and randomized controlled studies. In addition, retrospective studies of orthopedic trauma do not allow the determination of factors affecting implant selection. Fracture pattern, soft tissue involvement, and surgeon familiarity with implants may cause inherent selection bias that could potentially affect results.

## Conclusions

In this study, successful functional results were obtained in all three fixation methods applied to pediatric forearm double diaphyseal fractures. The hybrid fixation method was observed to be equal to the other methods or even advantageous in some aspects. Therefore, hybrid fixation can be used as a safe and effective method in appropriate pediatric forearm double diaphyseal fractures

## References

[1] Sinikumpu JJ, Lautamo A, Pokka T, Serlo W (2012). The increasing incidence of paediatric diaphyseal both-bone forearm fractures and their internal fixation during the last decade. Injury.

[2] Rennie L, Court-Brown CM, Mok JY, Beattie TF (2007). The epidemiology of fractures in children. Injury.

[3] Sun YQ, Penna J, Haralabatos SS, Carrion WV (2001). Intramedullary fixation of pediatric forearm diaphyseal fractures. Am J Orthop (Belle Mead NJ).

[4] Anderson LD, Sisk D, Tooms RE, Park WI 3rd (1975). Compression-plate fixation in acute diaphyseal fractures of the radius and ulna. J Bone Joint Surg Am.

[5] Schemitsch EH, Richards RR (1992). The effect of malunion on functional outcome after plate fixation of fractures of both bones of the forearm in adults. J Bone Joint Surg Am.

[6] Baldwin K, Morrison MJ 3rd, Tomlinson LA, Ramirez R, Flynn JM (2014). Both bone forearm fractures in children and adolescents, which fixation strategy is superior - plates or nails? A systematic review and meta-analysis of observational studies. J Orthop Trauma.

[7] Yao CK, Lin KC, Tarng YW, Chang WN, Renn JH (2014). Removal of forearm plate leads to a high risk of refracture: decision regarding implant removal after fixation of the forearm and analysis of risk factors of refracture. Arch Orthop Trauma Surg.

[8] Lascombes P, Prevot J, Ligier JN, Metaizeau JP, Poncelet T (1990). Elastic stable intramedullary nailing in forearm shaft fractures in children: 85 cases. J Pediatr Orthop.

[9] Garg NK, Ballal MS, Malek IA, Webster RA, Bruce CE (2008). Use of elastic stable intramedullary nailing for treating unstable forearm fractures in children. J Trauma.

[10] Westacott DJ, Jordan RW, Cooke SJ (2012). Functional outcome following intramedullary nailing or plate and screw fixation of paediatric diaphyseal forearm fractures: a systematic review. J Child Orthop.

[11] Zheng W, Tao Z, Chen C (2018). Comparison of three surgical fixation methods for dual-bone forearm fractures in older children: a retrospective cohort study. Int J Surg.

[12] Price CT, Scott DS, Kurzner ME, Flynn JC (1990). Malunited forearm fractures in children. J Pediatr Orthop.

[13] Marsh JL, Slongo TF, Agel J (2007). Fracture and dislocation classification compendium - 2007: Orthopaedic Trauma Association classification, database and outcomes committee. J Orthop Trauma.

[14] Schmittenbecher PP, Fitze G, Gödeke J, Kraus R, Schneidmüller D (2008). Delayed healing of forearm shaft fractures in children after intramedullary nailing. J Pediatr Orthop.

[15] Pace JL (2016). Pediatric and adolescent forearm fractures: current controversies and treatment recommendations. J Am Acad Orthop Surg.

[16] Zionts LE, Zalavras CG, Gerhardt MB (2005). Closed treatment of displaced diaphyseal both-bone forearm fractures in older children and adolescents. J Pediatr Orthop.

[17] Kang SN, Mangwani J, Ramachandran M, Paterson JM, Barry M (2011). Elastic intramedullary nailing of paediatric fractures of the forearm: a decade of experience in a teaching hospital in the United Kingdom. J Bone Joint Surg Br.

[18] Cullen MC, Roy DR, Giza E, Crawford AH (1998). Complications of intramedullary fixation of pediatric forearm fractures. J Pediatr Orthop.

[19] Jubel A, Andermahr J, Isenberg J, Issavand A, Prokop A, Rehm KE (2005). Outcomes and complications of elastic stable intramedullary nailing for forearm fractures in children. J Pediatr Orthop B.

[20] Ogonda L, Wong-Chung J, Wray R, Canavan B (2004). Delayed union and non-union of the ulna following intramedullary nailing in children. J Pediatr Orthop B.

[21] Fernandez FF, Eberhardt O, Langendörfer M, Wirth T (2009). Nonunion of forearm shaft fractures in children after intramedullary nailing. J Pediatr Orthop B.

[22] Flynn JM, Jones KJ, Garner MR, Goebel J (2010). Eleven years experience in the operative management of pediatric forearm fractures. J Pediatr Orthop.

[23] Reinhardt KR, Feldman DS, Green DW, Sala DA, Widmann RF, Scher DM (2008). Comparison of intramedullary nailing to plating for both-bone forearm fractures in older children. J Pediatr Orthop.

[24] Van der Reis WL, Otsuka NY, Moroz P, Mah J (1998). Intramedullary nailing versus plate fixation for unstable forearm fractures in children. J Pediatr Orthop.

[25] Zhu S, Yang D, Gong C, Chen C, Chen L (2019). A novel hybrid fixation versus dual plating for both-bone forearm fractures in older children: A prospective comparative study. Int J Surg.

[26] Feng Y, Shui X, Wang J, Cai L, Wang G, Hong J (2016). Comparison of hybrid fixation versus dual intramedullary nailing fixation for forearm fractures in older children: Case-control study. Int J Surg.

[27] Cai L, Wang J, Du S, Zhu S, Wang T, Lu D, Chen H (2016). Comparison of hybrid fixation to dual plating for both-bone forearm fractures in older children. Am J Ther.

